# Annotations

**Published:** 1894-05-05

**Authors:** 


### Annotations.

### Alcohol as a Physiological Peacemaker.

In a very learned paper read at the International Medical Congress on "The Ground-Substance of Protoplasm and its Modifications by Life," Dr. Danilewski, of St. Petersburg, made a decidedly novel statement which demands to be pondered by all-round abstainers from alcohol. Said Dr. Danilewski: "Civilised man uses alcohol so extensively, and has done so for so long, that one may with certainty affirm the existence of an alcoholised protoplasm in drunkards just as one finds morphinised protoplasm in cases of chronic intoxication with morphine. The existence of arsenic in the protoplasmic complex of arsenic-eaters, consequent on the fact that they are incapable of subsisting normally without that element, can no longer be questioned. In these three facts we have the proof that man by introducing into his body stimulant, narcotic, and alterative substances even, to excess becomes accustomed thereto to such a degree that without them his organism is not at peace." Of course the abstainer may very promptly answer this by asking the question, "Why should the organism be at peace?" And to this again the physiologist may reply, "Why should the organism continue to live?" As a matter of fact the organism as naturally seeks physiological contentment as it seeks to continue in life; and this fact must be reckoned with in any scheme of life or of dietary. The man who essays the conversion of his fellow men to total abstinence essays no smaller a task than the changing of the whole protoplasmic and material basis of the constitution of civilised man. In saying this we are not advancing an argument against total abstinence. We are merely inviting the abstaining propagandist to take a scientific and comprehensive view of the true nature of his task. If he still thinks the task desirable and himself competent, let him persevere in his mountain-levelling by all means.

### Diet and Stupidity.

"Some mothers distinctly diet their offspring for stupidity." This is Dr. Francis Walmsley's way of putting a physiological fact. Dr. Walmsley is medical superintendent of the Darenth Asylum, where he has 950 idiot, imbecile, and epileptic children under his care, and he makes his statement in his published annual report. Bad and unsuitable food, Dr. Walmsley tells us, often causes "thickening and enlargement of the skull"; and physiology has amply confirmed the old theory of our shoolboy days, that a thick skull is the outward and visible sign of dull and stupid brains. At Darenth very careful observations are made, and it is found that not only is the intellect poor in thick-skulled and rickety children, but the very senses themselves, the senses of sight, of hearing and of taste, and even the power of speech, are all remarkably impaired. Such children, Dr. Walmsley says, "see badly, hear badly, feel badly." In the matter of speech, the facts are almost startling. Of the 950 patients, only 360 articulate with normal distinctness. Of the remainder, 290 speak indistinctly, whilst 300 actually cannot speak at all; they are dumb. There is food for reflection here. We told the *Spectator* last week that the great public did not take in the discoveries and conclusions of medical science with any intelligent comprehension. As a matter of fact, no attempt whatever is made to deal with the tens of thousands of physiological facts of which reports like that of Dr. Walmsley are but indices. We need a physological revolution in our unscientific island. The apostles of science, unfortunately, have too often a poor opinion of what is nowadays called "altruism," and used to be called a good-natured interest in ones less fortunate neighbours. If only there could be found a score of enthusiastic physiologists possessed of a fine "enthusiasm of humanity," and with independent incomes and competent tongues and pens, what wonders might be wrought in a single decade. Dr. Walmsley deserves public thanks for his original and outspoken report.

### The Advancement of Science by Dining.

Everybody knows that hospitals "dine" themselves out of debt, but it has been reserved for a City company to discover the art of "dining" practical science up to a more rapid rate of progression. This is the feat by which the Worshipful Company of Plumbers is now succeeding in raising itself to influence and fame. The Plumbers' Company, being a very loyal company, were deeply moved by the spectacle, many years ago, of the Prince of Wales, who might be supposed to have all the resources of sanitary civilisation concentrated upon himself, lying sick unto death of so eminently preventable a disease as typhoid fever. No man, decided the Plumbers, who can live in a good house, ought to have typhoid fever. They went a step further. "In future," said they in effect, "no man, whether he lives in a good house or a bad one, shall have typhoid fever if we can prevent it." Now typhoid fever depends almost entirely upon the plumber, including always, of course, the water engineer. The Plumbers' Company forthwith set itself to secure a sound training in practice and an adequate education in principles for every plumber in the United Kingdom. The most effective step it could possibly take in that direction was to make it indispensable that any man who called himself a plumber should be registered by State authority, but only registered after furnishing proofs of competence in his art. From that day to this the Plumbers' Company has given its energies, its means, and its dinners for the furtherance of its great and worthy scheme of registration. On Wednesday week the company gave a dinner in the Fishmongers' Hall which was a fair specimen of all its dinners. The guests consisted of plumbers, doctors, sanitary engineers, members of Parliament interested in sanitation, *et hoc genus omne*. Now this is really a unique kind of spectacle—a City company devoting itself, its funds, even its dinners, with a strenuousness almost heroic, to so honourable an object as sanitation and the public health. The spectacle compels our sympathy. It ought to move even a great political party. For our part we appeal, first of all, to those non-sanitarian M.P.'s who block the Registration Bill to reconsider their position; and, secondly, to the cultivated and philosophical sanitarians on both sides of the House, of whom there are many, to rouse in themselves a little of the persevering enthusiasm of the Plumbers' Company, and to promptly pass into law a Bill so conducive in every way to the public well-being as the Plumbers' Registration Bill.

